# Role of Sport Activity on Quality of Life in Charcot-Marie-Tooth 1A Patients

**DOI:** 10.3390/jcm11237032

**Published:** 2022-11-28

**Authors:** Costanza Pazzaglia, Luca Padua, Claudia Stancanelli, Augusto Fusco, Claudia Loreti, Letizia Castelli, Isabella Imbimbo, Silvia Giovannini, Daniele Coraci, Gian Luca Vita, Giuseppe Vita

**Affiliations:** 1 UOC Neuroriabilitazione ad Alta Intensità, Fondazione Policlinico Universitario A. Gemelli IRCCS, 00168 Rome, Italy; 2 Department of Geriatrics and Orthopaedics, Università Cattolica del Sacro Cuore, 00168 Rome, Italy; 3 Nemo Sud Clinical Centre for Neuromuscular Disorders, 98125 Messina, Italy; 4 Department of Clinical and Experimental Medicine, Unit of Neurology, University of Messina, 98100 Messina, Italy; 5 Department of Aging, Neurological, Orthopaedic and Head-Neck Sciences, Fondazione Policlinico Universitario A. Gemelli IRCCS, 00168 Rome, Italy; 6 UOS Psicologia Clinica, Fondazione Policlinico Universitario A. Gemelli IRCCS, 00168 Rome, Italy; 7 UOS Riabilitazione Post-Acuzie, Fondazione Policlinico Universitario A. Gemelli IRCCS, 00168 Rome, Italy; 8 Department of Neuroscience, University of Padua, 35121 Padua, Italy; 9 Unit of Neurology, Department of Emergency, P.O. Piemonte, IRCCS Centro Neurolesi “Bonino-Pulejo”, 98124 Messina, Italy

**Keywords:** Charcot-Marie-Tooth, sport, quality of life, neuropathic pain, rehabilitation, personalized medicine

## Abstract

The present study aims to investigate the benefits induced by physical activity/practiced sport in Charcot-Marie-Tooth 1A (CMT1A). Patients were divided into sport and no-sport groups according to their sports performance habit. Thirty-one patients were enrolled, of which 14 practiced sports and 17 did not. Clinical assessments were administered to evaluate disability, self-esteem, depression, quality of life, and pain. Statistical analysis revealed significant differences in terms of gender in the no-sport group compared to the sport group (*p* = 0.04). Regarding the quality of life, physical function (*p* = 0.001), general health (*p* = 0.03), social function (*p* = 0.04), and mental health (*p* = 0.006) showed better patterns in the sport group than no-sport group. Moreover, neuropathic pain was reduced in the sport group according to the Neuropathic Pain Symptom Inventory (*p* = 0.001) and ID-PAIN (*p* = 0.03). The other administered questionnaires showed no significant differences. Our study confirms that CMT1A patients, who practice sports, with a similar severity of disability, may have a better physical quality of life while suffering less neuropathic pain than their peers who do not practice sports. Results recommend the prescription of sport in CMT1A patients.

## 1. Introduction

Sport is a valuable tool for improving the sense of wellbeing and quality of life, as well as reducing the social barriers of discrimination for disabled people [1]. Beyond the issue of individual autonomy, many physical, psychological, and social benefits derive from sports participation, which can result in reduced health service costs [2]. Several studies provide evidence about the protective role of physical activity in both physical and psychological domains [3,4,5]. The available evidence also reveals a positive impact of sport on areas of self-esteem, self-efficacy, and mental health [6,7].

Charcot-Marie-Tooth disease (CMT) is a hereditary sensory and motor neuropathy, characterized by genetic heterogeneity with a clinical phenotype of different severity, characterized by distal limb muscle deterioration and weakness, usually with skeletal deformities, distal sensory loss, and abnormalities of deep tendon reflexes [8].

The subdivision into demyelinating CMT1 and axonal CMT2 types is still valid for the majority of patients. CMT1A type represents 60–90% of CMT1 patients and 40–50% of all CMT patients [9].

Currently, there is no defined pharmacological therapy for this pathology. Past trials investigating the possible therapeutic efficacy of ascorbic acid in CMT failed [10,11,12,13]. A trial, in which the use of PXT3003 was tested, showed that this drug is safe and well tolerated, although its efficacy was reevaluated as a “slight” improvement in the erratum [14]. The other investigated molecules are currently involved in different phases of pharmacological testing, although their efficacy is still not proven in CMT1A patients [15].

Rehabilitation and surgical treatment, although without any defined protocol, remain to date the only options, while, for both interventions, there is no consensus about the best practice to recommend. Specifically, a common view lacks for rehabilitation programs and physical exercises to suggest despite general benefits of physical activity are known [16,17].

Sport could be a valid and more appealing integration to propose to these subjects, while also considering the early onset of syndrome. On the other hand, subjects affected by severe progressive genetic neuromuscular disease are usually considered by physicians and caregivers as unable to carry out sports activities, especially if a whole-body muscular effort is required. However, physicians arbitrarily recommend avoiding supramaximal exercise, when they rarely suggest patients to perform sports.

The purpose of this study was to analyze the impact of practiced sport on patients with CMT1A by investigating motivational effects, self-esteem, depression, quality of life, and pain, thus acquiring evidence on the role of sports as complementary therapy for CMT1A subjects.

## 2. Materials and Methods

In this study, 31 subjects affected by CMT1A were enrolled. Patients were divided into two groups: sport group (SG), which practiced sports for at least 6 months before the study onset for a frequency of at least 1 h/week and practiced with regularity; no-sport group (NSG), which did not currently practice sports. Inclusion criteria were as follows: (1) age > 18 years old; (2) clinical diagnosis and genetic confirmation of CMT disease; (3) Barthel index (BI) ≤90. Exclusion criteria were as follows: (1) inability to read, understand, and sign the informed consent form; (2) inability or unwillingness to complete the questionnaires; (3) having participated in clinical trials with experimental drugs in the 6 months prior to the study onset; (4) modification of pharmacologic therapy and rehabilitative treatment in the 6 months prior to the study; (5) hospitalization for acute events in the last 6 months; (6) sever concomitant diseases (i.e., cancer, cardiovascular and cerebrovascular diseases, etc.); (7) other conditions potentially affecting the peripheral nervous system (diabetes, vitamin deficiency, excessive alcoholic consumption, and foot surgery); (8) drug use with side-effects involving the peripheral nervous system.

CMT patients were enrolled in two different settings: outpatients consecutively evaluated for usual follow-up in dedicated clinical practice of highly specialized hospitals; members of Italian CMT patients’ social association (ACMT-Rete).

Data were obtained, anonymously, from patients who accepted to participate in the study and signed the informed consent. The study was conducted according to the Helsinki declaration on Ethical Principles for Medical Research Involving Human Subjects, and it was approved by the local ethics board (Prot. n. 5/26.12.2017_FDG). Questionnaires, as described below (included in Supplementary Materials), were administered in face-to-face modality or by telephonic interview. Due to the modalities of the interview, outcome measures needing clinical evaluation were not considered. Functional disability was assessed through the BI. BI evaluates disability in the activities of daily life and the level of dependence according to10 items, with a score ranging from 0 to 5 assigned to each. For this reason, the total score varies from a minimum of 0 to a maximum of 15. A score range from 0 to 24 shows a complete dependence level; a score range from 25 to 49 indicates severe dependence, a score range from 50 to 74 indicates moderate dependence, a score range from 75 to 90 indicates mild dependence, and a score range from 91 to 99 indicates minimal dependence [18]. Additionally, the Overall Neuropathy Limitations Scale (ONLS) was used to describe the limitations of upper and lower limbs [19].

### 2.1. Questionnaires

Initially, the patient’s sport practice level was investigated in order to assign subjects to SG or NSG according to the inclusion criteria. Consequently, the following topics were detected: (a) sport and motivational effects of physical activity performance; (b) self-esteem, depression, and quality of life; (c) pain.

#### 2.1.1. Sport and Motivational Effects of Physical Activity Performance

For the SG, data on sports habits were collected: (1) kind of sport; (2) length of period of practiced sport (in terms of months); (3) weekly frequency; (4) weekly timing of workout (min); (5) perceived benefits from sport; (6) participation in competitions. Moreover, data on available services were acquired: (1) facilities to practice sports in own neighborhood; (2) social network to engage sports and meetings among sports practitioners; (3) regularity; (4) perceived effort to practice.

For the evaluation of the motivational effect of physical activity, administered only to SG, Behavioral Regulation Exercise Questionnaire-2 (BREQ-2) was used. BREQ-2 includes 19 items, divided into different subclasses: amotivation, external regulation, introjected regulation, identified regulation, and intrinsic regulation. Responses were scored on a five-point scale ranging from 0 (“not true for me”) to 4 (“very true for me”). The mean of the five retrieved subscales was calculated on a five-point scale to evaluate the extent of each motivation type separately [20].

#### 2.1.2. Self-Esteem, Depression, and Quality of Life

Self-esteem was assessed through the Rosenberg Self-Esteem Scale (SES), a 10-item scale that measures global self-worth by measuring both positive and negative feelings about self: 10–16 is indicative of a low self-esteem; 17–33 is indicative of an adequate self-esteem; 34–40 is indicative of a high self-esteem [21].

Depressive symptomatology was evaluated through Beck Depression Inventory II (BDI-II), a self-report tool consisting of 21 items, divided into two factors: somatic–affective factor and cognitive factor of depression. The total score ranges between 0 and 63: 0–13 indicates a minimum depressive level; 14–19 indicates a medium level; 20–28 indicates a moderate level; 29–63 indicates a severe level of depressive symptoms [22].

Quality of life (QoL) was evaluated through the 36-item Short-Form Questionnaire (SF-36). This scale is divided into different subclasses (physical function; role physical; bodily pain; general health; vitality; social function; role of emotional and mental health), summarized in two main scores: physical composite score (PCS) and mental composite score (MCS). The total score was calculated using specific software [23]. The results were compared with the Italian normative data [24].

#### 2.1.3. Pain

The presence of pain, assessed in the whole sample (both SG and NSG), was investigated using the below-described questionnaires.

The neuropathic pain symptom inventory (NPSI) is a self-administered questionnaire specifically designed to evaluate the different symptoms of neuropathic pain. Developed and validated by Bouhassira and colleagues, it includes 12 items: ten descriptors of the different symptoms and two items for assessing the duration of spontaneous ongoing and paroxysmal pain. A total intensity score is calculated as the sum of the scores of the ten descriptors and five subscores that are calculated through the mean scores of the items belonging to each of the five factors identified in the factor analysis [25]. We used the validated Italian version of the NPSI [26].

ID-PAIN is a six-item self-administered questionnaire whose score ranges from 0 to 5, with higher scores corresponding to neuropathic pain or mixed pain with neuropathic component. The presence of pain limited to the joints (i.e., nociceptive pain) is scored minus 1. A score of −1 means “improbable neuropathic pain”, 1 means “possible neuropathic pain”, 2–3 mean “probable neuropathic pain”, and 4–5 mean “high probable neuropathic pain” [27].

### 2.2. Statistical Analisys

Descriptive analysis assessing the general characteristics of the whole sample and of each group was performed. Clinical outcomes were analyzed by nonparametric statistical analysis. Between-group comparisons were evaluated using the Mann–Whitney U test or, in the case of dichotomous measurements, a 2 × 2 table chi-square test. The significance level was set at *p* < 0.05.

## 3. Results

Thirty-one patients affected by CMT1A were enrolled; 14 were considered sport practitioners and 17 were not on the basis of their sports activity for at least 6 months before the study onset. Demographic characteristics and data on disability were collected and are reported in Table 1. The number of females in NSG was higher compared to SG (*p* = 0.04). No significant differences were found in terms of disability evaluated by BI by statistical between-group analysis (Table 1).

With regard to the data on sports activity collected in the SG, 67% of patients practiced gym, 13% practiced swimming, 7% practiced yoga, 7% practiced boating, and 6% practiced horse riding. None of the subjects trained in two or more sports disciplines contemporarily during the period considered in the study. Sports were practiced for 102 months on average (range: 12–360; mean value: 102; SD: ±111.7), with a mean frequency of 2.7 times per week (range: 1–5; mean value: 2.7; SD: ±1.2), for 72.9 min of mean workout time (range: 60–100; mean value: 72.9; SD: ±22.7); weekly minutes of workout were 205.7 on average (range: 60–600 min; mean value: 205.7; SD: ±141.1). No study participants took part in any sports competitions throughout or before the study period. All patients declared being easily able to perform sports in their own neighborhood; information on sports facilities and related meetings was equally easy to obtain.

Referring to participation level regularity, 49% of patients practiced “enough”, whereas 29% were “high”, 14% were “normal”, and 14% were “low”. Table 2 shows the results of the BREQ-2 assessment of the motivational effects of physical activity, administered only to the SG, while the SES showed an adequate to high level of self-esteem in the entire sample, and an overall minimal depressive level was assessed by the BDI-II.

The QoL was lower in the whole sample compared to data presented by Italian normative values, as measured by the SF-36. Furthermore, better patterns were revealed in SG than NSG in the following physical scores: physical function (*p* = 0.001), general health (*p* = 0.03), social function (*p* = 0.04), and mental health (*p* = 0.006) (Table 3).

Pain was represented as a potentially neuropathic symptom and characterized by low intensity. Pain in SG resulted less neuropathic according to NPSI and ID-PAIN (*p* = 0.001 and *p* = 0.03, respectively), as reported in Table 4.

## 4. Discussion

The study results showed that CMT1A patients who regularly practice sports had a better physical QoL and presented less neuropathic pain compared to the non-sport-practicing subjects [28,29]. Several studies investigated the variables influencing quality of life [30,31,32,33,34], such as increased fatigue [35], disability [36], muscle weakness [37,38], poor sensory function, and skeletal abnormalities [39,40]. Our data showed that QoL was more negatively influenced in NSG patients in comparison with SG patients. Specifically, differences emerged from the subitems physical function, general health, social function, and mental health. These data could be interpreted as a link between the ability to perform sport and the mildness of symptoms. This should be additionally associated with the potential effects of physical condition in social life among the sportsmen. Hackett et al. showed that, in CMT patients, QoL is probably also influenced by body mass. In particular, lower mass is associated with a better physical performance in CMT patients [41]. Thus, the advantage of sport could be related to its positive impact in weight control. This hypothesis is also supported by Roberts-Clarke et al. which investigated physical performance variables associated with better QoL in CMT; higher leg press power, faster habitual gait speed, left hip abduction, and seated row strength were related to higher physical function [42]. Similar results were obtained in our previous findings using a wearable accelerometer, reflecting daily activity parameters; outputs reflecting higher speed of walking and higher explosive performance correlated with better physical QoL, while a lower number of daily steps correlated with higher deterioration of QoL [43].

Concerning the age difference between SG and NSG, although not statistically significant, it is known that CMT is a pathology characterized by a childhood onset and a progressive degeneration over time; hence, it is possible to hypothesize that the better outcome of the SG is mostly due to the younger age rather than to the beneficial effect of the sport. Indeed, an article by Tozza et al. [44] pointed out the age of 50 years as a crucial age beyond which patients showed a significant motor impairment. Both groups included patients older than 50 years; thus, we think the results could be due mostly to the beneficial effects of the sport instead of age differences.

Moreover, the groups also differed according to the gender distribution; we hypothesized that the different distribution between male and female in the two groups could be explained by the fact the females experienced the symptoms and the limitations induced by the pathology earlier than males. This is in accordance with a previous study performed by Colomban et al. [45], in which the authors found, in a large court of CMT1 A patients, that onset of symptoms was earlier in women compared to men. Moreover, upper- and lower-limb disability (including difficulties in running and jumping, and falls) was higher in women, reflecting a higher deterioration in QoL. This could explain why females had a less frequent habit of practicing sport.

With respect to pain, although it is not a hallmark symptom of the disease, it is an impactful symptom for patients suffering from it. There are still several gaps concerning the knowledge of pain in this pathology, e.g., its origin. A past study performed by our group showed that CMT1A patients complain not only about nociceptive but also about neuropathic pain, probably because of an involvement of A-delta fibers, as confirmed by a laser-evoked experiment [46]. The possible neuropathic origin of pain was also confirmed by the study of Ribiere et al. [47], assessed by specific neuropathic pain questionnaires, while Laurà et al., by administering pain-specific questionnaires and performing a study of small fibers, found a combination of neuropathic and nociceptive pain [48]. In our sample, the comparison of SG and NSG showed that the latter suffered more from neuropathic pain than the former. This allows us to speculate that sport reduces the presence of pain in sport-practicing patients or that high neuropathic pain is a limiting factor for sport practice. Indeed, a study conducted by Anens and colleagues found that pain, fatigue, poor balance, and muscle weakness are important obstacles to physical activity [49], thus highlighting the effect of pain as a limiting physical factor. According to the study conducted by Azevedo and colleagues, pain is also a symptom with a negative influence on the patient’s life, thus limiting the activities of daily life and sports due to both physical and emotional suffering. For this reason, the patient often feels limited and prefers rest and sedentary life to any type of activity [50].

On the other hand, studies performed in the field of physical activity as a possible therapy for pain showed that exercises at a high performance also determine an overall improvement of physical function including pain [51].

Although the current results did not allow us to determine if pain is a limiting factor to practice sport or if it is a symptom that benefits from sport practice, they showed that sport has a positive influence on the disease and physical exercise does not give a harmful effect on patients with CMT. These results confirms the study of Piscosquito and colleagues, whose objective was to determine if the overwork hypothesis (OW) was true for CMT [52]. The authors’ data do not support the OW hypothesis and the consequent harmful effect of the exercise in patients with CMT1A. In CMT1A, the weakness is due to the disease itself, and OW produces no further weakness of the overloaded muscles; therefore, patients with CMT should not limit limb use in everyday life to prevent loss of muscle strength because OW has no role in the progression of the disease [52]. This was confirmed by Tajima et al. [53], who affirmed that “progressive resistance strengthening programs for lower extremities are feasible, safe, and beneficial, and improve exercise intolerance and undue fatigue in patients with CMT. Although the improvement in exercise tolerance may be partly due to the reversal of deconditioning effect of related sedentary lifestyle, progressive resistance training and physical fitness can improve walking function, activities of daily living, and subjective perception of pain and fatigue in patients with CMT” [53].

The study results are in line with Vita et al. [54], reporting the positive physical, emotional, and psychosocial changes induced by sport activity in a Paralympic swimmer with CMT4A. The authors concluded that an intensive muscular training induced a marked improvement of QoL, removal of depression, and reduction in trait anxiety. Thanks to sport practice, the patients experienced increased self-esteem and self-efficacy. Moreover, Vita et al. reported, in a study conducted on patients affected by muscular disease of different etiology, that patients who practiced sport had a better outcome in terms of self-esteem, depression, greater social identity, adherence, and QoL with respect to those that do not practice sport [3,55,56]. Similarly to our results and conclusion, those authors suggest recommending sport activity in patients affected by neuromuscular disorders, as a complementary therapy able to improve mental and social wellbeing.

The main limitations of this study are its small sample size, which may be explained by the rare nature of the disease. Moreover, CMT1A patients suffer from a lack of pharmacological therapies and a scarce knowledge of their disease by physicians and healthcare professionals. These are some of the factors that can explain the reduce compliance of patients to respond to a battery of several questionnaires.

Furthermore, in order to respect the almost 1:1 ratio between SG and NSG, during the enrollment of patients, we had difficulties in recruiting CMT1A patients that practice some sports because patients are afraid that sports could cause a worsening of their conditions. The importance of this paper is also in disproving this belief. Another important weak point is the absence of randomization, which did not allow understanding the real reasons for the differences between the two groups. In particular, some subjects did not practice sport because of their major impairments or their lower psychological health. Moreover, the main specific scale, Charcot-Marie-Tooth Neuropathy Score (CMT-NS), was not applied because some of the patients were interviewed by telephone. However, the results seem to be consistent and statistically clear. Further studies, perspective and randomized, involving other large, specialized centers, can overcome the important limitations of our present work.

## 5. Conclusions

Our study confirmed that patients who performed sport, presenting a similar disability, had a better physical QoL and less neuropathic pain compared to those who did not perform sport. These results encourage the prescription of sport in CMT-1A patients and suggest prospective studies in this field.

## Figures and Tables

**Table 1:** Demographical and disability data of the whole sample and in the sport group and no-sport group.

		Whole Sample (n = 31)	SG (n = 14)	NSG (n = 17)	*p*
**Age (years)**	(mean ± SD) Range	45.5 ± 18.9 18–79	39.6 ± 21.9 18–79	50.4 ± 15.0 18–70	0.31
**M/F**		17/14	11/3	6/11	**0.04**
**BI**	(mean ± SD) Range	82.7 ± 4.0 73–85	84.7 ± 0.7 83–85	81.0 ± 4.8 73–85	0.27
**ONLS**	(mean ± SD) Range	6.2 ± 1.1 3–8	6.3 ± 1.3 3–8	6.2 ± 1.2 3–8	0.21

SG: sport group; NSG: no-sport group; BI: Barthel index; ONLS: Overall Neuropathy Limitation Scale; SD: standard deviation. Significance level: *p* < 0.05 (in bold).

**Table 2:** Motivational effects of physical activity performance, depression, and self-esteem of whole sample and for each group.

		Whole Sample (n = 31)	SG (n = 14)	NSG (n = 17)	*p*
**BREQ-2 AM**	(mean ± SD)	-	0.04 ± 0.13	-	
**BREQ-2 ER**	(mean ± SD)	-	0.23 ± 0.44	-	
**BREQ-2 INR**	(mean ± SD)	-	1.07 ± 0.75	-	
**BREQ-2 IDR**	(mean ± SD)	-	2.98 ± 0.09	-	
**BREQ-2 INTRIN TOT**	(mean ± SD)	-	2.66 ± 0.35	-	
**BDI-II**	(mean ± SD)	6.03 ± 6.65	5.14 ± 6.92	6.76 ± 6.53	0.11
**SES**	(mean ± SD)	33.16 ± 4.95	33.64 ± 4.66	32.76 ± 5.28	0.32

SG: sport group; NSG: no-sport group; BREQ-2: Behavioral Regulation Exercise Questionnaire-2 (for the sport group only); AM: amotivation; ER: external regulation; INR: introjected regulation; IDR: identified regulation; INTRIN TOT: intrinsic regulation; BDI-II: Beck Depression Inventory-II; SES: Rosenberg Self-Esteem Scale; SD: standard deviation. Significance level: *p* < 0.05.

**Table 3:** Quality of life data of the whole sample and in sport and no-sport group, assessed by SF-36.

	Whole Sample (n = 31)	SG (n = 14)	NSG (n = 17)	*p*
**Physical function**	61.3	83.9	42.6	**0.001**
**Role physical**	69.4	83.9	57.4	0.33
**Bodily pain**	72.2	83.7	62.8	0.22
**General health**	52.8	61.3	45.8	**0.03**
**Vitality**	63.1	66.8	60.0	0.17
**Social function**	77.8	86.6	70.6	**0.04**
**Role emotional**	80.6	88.1	74.5	0.09
**Mental health**	72.0	74.9	69.6	**0.006**
**PCS**	42.1	49.6	35.8	0.22
**MCS**	51.7	51.6	51.8	0.18

SG: sport group; NSG: no-sport group; PCS: physical component summary; MCS: mental component summary. Significance level: *p* < 0.05 (in bold).

**Table 4:** Pain of the whole sample and in sport and no-sport groups.

		Whole Sample (n = 31)	SG (n = 14)	NSG (n = 17)	*p*
**NPSI**	(mean ± SD)	13.2 ± 18.4	2.1 ± 4.0	22.3 ± 20.6	**0.001**
**ID pain**	(mean ± SD)	2.0 ±1.2	0.1 ± 0.4	1.4 ± 1.5	**0.03**

SG: sport group; NSG: no-sport group; NPSI: Neuropathic Pain Symptom Inventory; SD: standard deviation. Significance level: *p* < 0.05 (in bold).

## Data Availability

Not applicable.

## Supplementary Materials

The following supporting information can be downloaded at: https://www.mdpi.com/article/10.3390/jcm11237032/s1, Questionnaires. Reference [57] is cited in the Supplementary Materials.
