# Supplementary material for: Role of Sport Activity on Quality of Life in Charcot-Marie-Tooth 1A Patients
Source: J Clin Med. 2022 Nov 28;11(23):7032. doi: 10.3390/jcm11237032 (PMC9740468; doi:10.3390/jcm11237032)
Supplement: Supplementary file 1 [file jcm-11-07032-s001.zip › BDI.pdf]

BECK DEPRESSION INVENTORY

Nel questionario vi sono gruppi di dichiarazioni. Legga con attenzione ciascun gruppo e da ognuno scelga la dichiarazione che meglio descrive il modo in cui si è sentito durante l'ultima settimana, oggi compreso.

**A**	□ (0) Non mi sento triste<br>□ (1) Mi sento triste<br>□ (2) Sono sempre triste e non riesco a star bene<br>□ (3) Sono così triste e infelice che non riesco a sopportarlo
**B**	□ (0) Non sono particolarmente scoraggiato per il futuro<br>□ (1) Mi sento scoraggiato per il futuro<br>□ (2) Ho la sensazione di non desiderare nulla intensamente<br>□ (3) Ho la sensazione che il futuro è senza speranza e che le cose non possano migliorare
**C**	□ (0) Non mi sento un fallito<br>□ (1) Ho la sensazione di aver fallito più di una persona media<br>□ (2) Se ripenso alla mia vita riesco a vedere solo una serie di fallimenti<br>□ (3) Ho la sensazione di essere un fallimento totale come persona
**D**	□ (0) Dalle cose traggo la stessa soddisfazione di prima<br>□ (1) Non mi godo le cose come facevo un tempo<br>□ (2) Non traggo più soddisfazione da nulla<br>□ (3) Sono insoddisfatto o annoiato di tutto
**E**	□ (0) Non mi sento particolarmente colpevole<br>□ (1) Mi sento colpevole per buona parte del tempo<br>□ (2) Mi sento colpevole per la maggior parte del tempo<br>□ (3) Mi sento sempre colpevole
**F**	□ (0) Non ho la sensazione di star subendo una punizione<br>□ (1) Ho la sensazione che potrei subire una punizione<br>□ (2) Mi aspetto di essere punito<br>□ (3) Ho la sensazione di star subendo una punizione
**G**	□ (0) Non mi sento deluso di me stesso<br>□ (1) Sono deluso di me stesso<br>□ (2) Sono disgustato di me stesso<br>□ (3) Odio me stesso
**H**	□ (0) Non credo di essere peggiore di chiunque altro<br>□ (1) Mi critico per le mie debolezze o per i miei errori<br>□ (2) Mi rimprovero continuamente per le mie colpe<br>□ (3) Mi rimprovero per tutte le cose brutte che accadono
**I**	□ (0) Non penso mai ad uccidermi<br>□ (1) Mi vengono idee di uccidermi ma non le realizzerei mai<br>□ (2) Vorrei uccidermi<br>□ (3) Mi ucciderei se potessi
**L**	□ (0) Non piango più del solito<br>□ (1) Ora piango più che in passato<br>□ (2) Ora piango continuamente<br>□ (3) Un tempo riuscivo a piangere, ma ora non ci riesco anche se ne ho voglia

**M**	◻ (0) Non sono più irritato del solito<br>◻ (1) Mi infastidisco e mi irrito più facilmente di un tempo<br>◻ (2) Ora sono continuamente irritato<br>◻ (3) Non mi irrito affatto per le cose che un tempo mi irritavano

**N**	◻ (0) Non ho perso interesse per le altre persone<br>◻ (1) Ora ho meno interesse che nel passato per le altre persone<br>◻ (2) Ho perso la maggior parte dell'interesse per le altre persone<br>◻ (3) Ho perso completamente interesse per le altre persone

**O**	◻ (0) Prendo decisioni quasi come al solito<br>◻ (1) Cerco di rimandare le decisioni più spesso di un tempo<br>◻ (2) Ho maggiore difficoltà di un tempo a prendere decisioni<br>◻ (3) Non riesco più a prendere alcuna decisione

**P**	◻ (0) Non credo di avere un aspetto peggiore del solito<br>◻ (1) Sono preoccupato di apparire vecchio o non attraente<br>◻ (2) Sento che il mio aspetto ha subito cambiamenti permanenti che mi rendono meno attraente<br>◻ (3) Ho la sensazione di essere brutto

**Q**	◻ (0) Riesco a lavorare quasi altrettanto bene che in passato<br>◻ (1) Devo sforzarmi di più per cominciare a fare qualcosa<br>◻ (2) Devo spronare fortemente me stesso per fare qualunque cosa<br>◻ (3) Non riesco a lavorare per niente

**R**	◻ (0) Dormo bene come al solito<br>◻ (1) Non dormo bene come prima<br>◻ (2) Mi sveglio una o due ore prima del solito e mi riesce difficile riaddormentarmi<br>◻ (3) Mi sveglio parecchie ore prima del solito e non riesco più ad addormentarmi

**S**	◻ (0) Non mi sento più stanco del solito<br>◻ (1) Mi stanco più facilmente di un tempo<br>◻ (2) Mi stanco a fare qualunque cosa<br>◻ (3) Sono troppo stanco per fare alcunché

**T**	◻ (0) Non ho meno appetito del solito<br>◻ (1) Non ho un buon appetito come una volta<br>◻ (2) Ora ho molto meno appetito<br>◻ (3) Non ho più appetito per niente

**U**	◻ (0) Di recente non sono calato molto di peso<br>◻ (1) Ho perso più di due chili<br>◻ (2) Ho perso più di quattro chili<br>◻ (3) Ho perso più di sei chili

**V**	◻ (0) Non mi preoccupo della mia salute più del solito<br>◻ (1) Mi preoccupo per le fitte e i dolori o per il mal di stomaco o per la stitichezza<br>◻ (2) Mi preoccupo talmente di come mi sento che mi è difficile pensare ad altro<br>◻ (3) Sono così preoccupato di come mi sento che non penso ad altro

**Z**	◻ (0) Non ho notato di recente alcun cambiamento nel mio interesse per il sesso<br>◻ (1) Ho meno interesse di una volta per il sesso<br>◻ (2) Ho molto meno interesse per il sesso<br>◻ (3) Ho perso completamente interesse per il sesso
