# Supplementary material for: Role of Sport Activity on Quality of Life in Charcot-Marie-Tooth 1A Patients
Source: J Clin Med. 2022 Nov 28;11(23):7032. doi: 10.3390/jcm11237032 (PMC9740468; doi:10.3390/jcm11237032)
Supplement: Supplementary file 1 [file jcm-11-07032-s001.zip › BREQ-2_pdf.pdf]

BEHAVIOURAL REGULATION IN EXERCISE QUESTIONNAIRE-2 (BREQ-2)

Markland & Tobin (2004) - Costa, Oliva, Cuzzocrea, & Larcan (2013)

Siamo interessati ad indagare le ragioni che spingono molte persone a impegnarsi nella pratica dell'esercizio fisico. Utilizzando la scala di seguito, la preghiamo di indicare in quale misura ciascuna delle seguenti affermazioni sia vera per lei. Non ci sono risposte giuste o sbagliate. Vogliamo semplicemente conoscere la sua opinione circa la pratica sportiva. Le sue risposte saranno trattate con la massima riservatezza e utilizzate esclusivamente per scopi di ricerca.

		**Non vero**	**Qualche volta vero**		**Molto vero**
**1**	Faccio attività fisica perché gli altri dicono che dovrei farlo	0	1	2	3
**2**	Mi sento in colpa quando non faccio esercizio fisico	0	1	2	3
**3**	Apprezzo i benefici dell'attività fisica	0	1	2	3
**4**	Faccio attività fisica perché è divertente	0	1	2	3
**5**	Non riesco a capire il motivo per cui dovrei fare attività fisica	0	1	2	3
**6**	Faccio esercizio fisico perché i miei amici/familiari/ partner affermano che dovrei	0	1	2	3
**7**	Mi vergogno quando salto una sessione di allenamento	0	1	2	3
**8**	Per me è importante fare esercizio fisico con regolarità	0	1	2	3
**9**	Non riesco a capire il motivo per cui dovrei preoccuparmi di fare attività fisica	0	1	2	3
**10**	Mi diverto mentre faccio attività fisica	0	1	2	3
**11**	Faccio attività fisica perche gli altri non sarebbero contenti di me se non lo facessi	0	1	2	3
**12**	Non vedo la ragione per cui fare attività fisica	0	1	2	3
**13**	Mi sento depresso quando non mi alleno anche per un breve periodo di tempo	0	1	2	3
**14**	Credo sia importante fare lo sforzo di allenarsi regolarmente	0	1	2	3
**15**	Ritengo che fare esercizio fisico sia una attività molto piacevole	0	1	2	3
**16**	Mi sento sotto pressione da parte dei mie amici/familiari a fare attività fisica	0	1	2	3
**17**	Divento inquieto se non mi alleno con regolarità	0	1	2	3
**18**	Traggo piacere e soddisfazione nel fare esercizio fisico	0	1	2	3
**19**	Penso che fare attività fisica sia una perdita di tempo	0	1	2	3

BREQ-2

Amotivation	5	9	12	19
External regulation	1	6	11	16
Introjected regulation	2	7	13	
Identified regulation	3	8	14	
Intrinsic regulation	4	10	15	18

FREQUENZA DELL'ESERCIZIO FISICO

1) Generalmente quanti giorni a settimana ti alleni?

2) Generalmente, quante ore al giorno ti alleni?
