# Supplementary material for: Role of Sport Activity on Quality of Life in Charcot-Marie-Tooth 1A Patients
Source: J Clin Med. 2022 Nov 28;11(23):7032. doi: 10.3390/jcm11237032 (PMC9740468; doi:10.3390/jcm11237032)
Supplement: Supplementary file 1 [file jcm-11-07032-s001.zip › ID PAIN.pdf]

ID-PAIN QUESTIONARIO

PARTECIPANTE ……………………………………………………………………………………

DATA …………………………………………………………….

SEGNARE "**SI"** NEI CAMPI CHE CORRISPONDONO AL DOLORE PROVATO NELL'

ULTIMA SETTIMANA E "**NO**" IN QUELLI CHE NON CORRISPONDONO:

DOMANDE:

1. Il dolore era simile a punture di aghi e spilli? SI (1) NO (0)

2. Il dolore era di tipo bruciante/urente? SI (1) NO (0)

3. Il dolore era di tipo addormentamento? SI (1) NO (0)

4. Il dolore era simile ad una scossa elettrica? SI (1) NO (0)

5. Il dolore peggiora al tatto con i vestiti o le lenzuola? SI (1) NO (0)

6. Il dolore è limitato alle articolazioni? SI (-1) NO (0)
