# Supplementary material for: Role of Sport Activity on Quality of Life in Charcot-Marie-Tooth 1A Patients
Source: J Clin Med. 2022 Nov 28;11(23):7032. doi: 10.3390/jcm11237032 (PMC9740468; doi:10.3390/jcm11237032)
Supplement: Supplementary file 1 [file jcm-11-07032-s001.zip › NPSI.pdf]

Neurol Sci (2009) 30:99–106
DOI 10.1007/s10072-009-0025-y

ORIGINAL ARTICLE

Validation of the Italian version of the Neuropathic Pain Symptom Inventory in peripheral nervous system diseases

Luca Padua · Chiara Briani · Stefano Jann · Eduardo Nobile-Orazio · Costanza Pazzaglia · Alberto Morini · Mauro Mondelli · Palma Ciaramitaro · Guido Cavaletti · Dario Cocito · Raffaella Fazio · Lucio Santoro · Francesca Galeotti · Marinella Carpo · Rosaria Plasmati · Luana Benedetti · Angelo Schenone · Paolo Marchettini · Giorgio Cruccu

Received: 2 July 2008 / Accepted: 24 December 2008 / Published online: 6 February 2009
© Springer-Verlag 2009

Abstract The aim of this study was to validate the Italian version of the Neuropathic Pain Symptom Inventory (NPSI) in patients with neuropathic pain due to peripheral nerve diseases, and also to evaluate the validity of a new NPSI score: a frequency weighted NPSI score (NPSI-FW). First, the original version of the NPSI was translated into Italian. Then the validity and reliability of the Italian NPSI (I-NPSI) were tested in 392 Italian patients consecutively referred to 16 Italian outpatient services for peripheral nerve diseases, by correlating the I-NPSI scores with other pain scales. The repeatability and responsiveness were assessed. A significant correlation between the I-NPSI scores and all the other

L. Padua (✉) · C. Pazzaglia
Department of Neurosciences, Institute of Neurology,
UCSC University, Largo F. Vito 1, 00168 Rome, Italy
e-mail: lpadua@rm.unicatt.it

L. Padua · C. Pazzaglia
Don Gnocchi Foundation, Rome, Italy

C. Briani
Department of Neurosciences, University of Padua, Padua, Italy

S. Jann
Department of Neurology, Neuromuscular Unit,
Niguarda Hospital, Milan, Italy

E. Nobile-Orazio
Department of Neurological Sciences,
2nd Neurology, IRCCS Humanitas Clinical Institute,
Milan University, Rozzano, Milan, Italy

A. Morini
Department of Neurology, Neurophysiology Unit,
Ospedale Santa Chiara, Trento, Italy

M. Mondelli
EMG Service, ASL 7, Siena, Italy

P. Ciaramitaro
Clinical Neurophysiology, Neurosurgery Division,
CTO Hospital, Torino, Italy

G. Cavaletti
Department of Neurosciences and Biotechnology,
Milan University, Monza, Italy

D. Cocito
Clinical Neurophysiology Unit, Neurosciences Department,
S. Giovanni Battista Hospital, Torino, Italy

R. Fazio
Department of Neurology, IRCCS San Raffaele Hospital,
Milan, Italy

L. Santoro
Scienze Neurologiche, Università Federico II di Napoli,
Naples, Italy

F. Galeotti
Department of Neurological Sciences,
La Sapienza University, Rome, Italy

M. Carpo
Department of Neurological Sciences, Italy IRCCS,
Fondazione Policlinico, Milan University, Milan, Italy

R. Plasmati · G. Cruccu
Department of Neurosciences, Bellaria Hospital, Bologna, Italy

L. Benedetti
Department of Neurology, Osp. S. Andrea, La Spezia, Italy

A. Schenone
Department of Neurosciences, Ophthalmology and Genetic,
University of Genova, Genova, Italy

P. Marchettini
Pain Medicine Center, Scientific Institute San Raffaele, Clinica
San Carlo, Paderno Dugnano, MI, Italy

pain measures was seen. Reproducibility and responsiveness were good. Our study shows the validity of the I-NPSI and demonstrates its reliability for assessing neuropathic pain in patients with peripheral nerve diseases. The I-NPSI scores represent reliable measurements to assess neuropathic symptoms and effectiveness of treatment on them.

Keywords Questionnaire · Validation · Neuropathic pain · Pain · Patient-oriented · Peripheral nervous system

Introduction

Patient-oriented measures using self-administered questionnaires have added a new dimension to clinical outcome evaluation [1–3]. The most commonly used tools to assess the patient's perspective are self-administered questionnaires. Pain is a subjective condition that cannot be objectively measured; for this reason self patient-perspective is fundamental.

Neuropathic Pain Symptom Inventory (NPSI) was designed to evaluate the different symptoms of neuropathic pain [4]. The original study showed that NPSI allows discrimination and quantification of five distinct clinically relevant dimensions of neuropathic pain, and that it is able to measure changes due to treatment. The self-administered questionnaires must be subjected to a thorough validation process to evaluate their reliability and validity [5]. Thus, for the purpose of using the NPSI as outcome measure in multi-center international studies, this questionnaire must be translated and culturally adapted in order to enable its use with different language groups and in different countries. It must then be validated against the original version. The cross-cultural adaptation guidelines described by Guillemin et al. [5] are widely accepted and used for the translation and adaptation of questionnaires. We followed these guidelines for the trans-cultural adaptation and validation of the Italian version of the NPSI. The second aim of the study was to evaluate the validity of a new NPSI score assessing the temporal pattern of pain.

Patients and methods

In accordance with previously published guidelines [5] we submitted the NPSI questionnaire to the validation process after translation and cultural adaptation for use in an Italian speaking population. The steps for validation are described as follows:

Translation

A professional English–French translator, whose first language is Italian, initially translated the original (French) and English NPSI into Italian. Two physicians, whose first language is Italian, made another translation from the English and French versions into Italian. As recommended by Guillemin et al. [5], a back-translation into English and French was then performed, in order to check for inconsistencies with the original text.

The Italian version of the NPSI (I-NPSI) was analyzed for Italian cultural characteristics and no inconsistencies were observed.

Patients

The final version of the questionnaire was administered to 392 Italian patients (men/women 218/174; mean age 58.8, range 16–89) with peripheral nerve diseases referring to 16 Italian neurological centers. Peripheral nerve disease diagnosis was based on clinical features supported by neurophysiology study, neuroimaging, and blood tests according to clinician's indication. Essential clinical diagnostic criteria were the presence of sensory or motor deficits with a radicular, troncular or distally symmetric distribution and reduced, or absent deep tendon reflexes. Neurophysiological studies helped define the presence of radiculopathies, entrapment neuropathies, mononeuritis multiplex, polyneuropathies, or plexopathies. Neuroimaging often supported the radiculopathy findings. History of alcohol abuse or exposition to neurotoxic drugs (e.g. chemotherapies) was carefully investigated. The presence of diabetes or impaired glucose intolerance, together with the most common causes of neuropathies was recorded. Antibody to peripheral nerve antigens helped to identify autoimmune neuropathies. Based on clinical, neurophysiological, and biochemical data, the patients were classified as affected by in different types of peripheral nervous system involvement, as summarized in Table 2.

Inclusion criteria were consecutive patients clinically affected by a diffuse or focal damage of peripheral nervous system, regardless of pain. Exclusion criteria were pain not due to peripheral nerve diseases (e.g. bone metastasis, arthritis), cognitive impairment (Mini Mental State <24), age less than 16 years and psychiatric disorders. Pain medications did not represent an exclusion criterion. The following clinical data were collected: age, gender, clinical history (specially focused on pain), diagnosis, drugs, and duration of pain (if present). I-NPSI items for which patients requested help and the mean duration for filling in the questionnaire were collected. Table 1 shows the characteristics of the Italian and original patient populations included in the study by Bouhassira et al. [4]. Table 2 shows the diagnosis of the enrolled population.

Outcome tools

NPSI includes 12 items: ten descriptors of the different symptoms and two items for assessing the duration of

Table 1 Descriptive statistics of Italian and French patient populations

Parameter	Italian population $n = 392$	French population $n = 176$	*P* value
Men/women	218/174	97/79	NS
Mean age (range)	58.8 (16–89) (SD: 15.7)	55 (20–85)	NS[b]
Mean duration of pain, months (range)	29.5 (1–360) (SD: 44.3)	70 (6–420)[a]	<0.0001[b]
Mean pain intensity (VAS) (range)	37.2 (0–100) (SD: 29.3)	65 (30–100)[a]	<0.0001[b]

SD standard deviation, *P P* value, *NS* non significant

[a] The inclusion criterion was the presence of pain with VAS at least ≥30

[b] *t* test assuming that the SD of French population is the same of Italian population because it is not provided in the original study

Table 2 Etiology of peripheral nerve involvement

	Percentage
Non-diabetic polyneuropathy	51.8
Diabetic polyneuropathy	13.4
Entrapment	12.1
Radiculopathies	8.3
Neuropathies multiplex	6.4
Traumatic nerve injury	6.2
Other	1.8

spontaneous ongoing and paroxysmal pain. A total intensity score is calculated as the sum of the scores of the ten descriptors and five subscores (Table 3) that are calculated through the mean scores of the items belonging to each of the five factors identified in the factor analysis. In the original version of the NPSI the two items for assessing temporal pattern of pain are not included in the final score. We propose to include these items in the final score, obtaining a frequency weighted score (NPSI-FW); note that, because of the original temporal items structure, contrary to the other scores, the best situation is 0 and the worst is 5.

The Italian version of the NPSI is presented in "Appendix".

The following outcome measures for construction validity were adopted:

- Douleur Neuropathique 4 (DN4) and ID pain in order to distinguish between the presence and absence of neuropathic pain [6, 7].
- Visual Analog Scale (VAS) in order to score entity of pain in the last 24 h [8].

For the VAS, we asked the patients to mark the level of their pain on a 100-mm line marked at one end as "no pain" and at the other as "worst pain imaginable".

DN4 is a clinician-administered questionnaire consisting of ten items: seven items concern the quality of pain, are obtained by interviewing the patients, whereas three items are based on clinical examination and analyze the presence or absence of touch or pinprick hypoesthesia and tactile allodynia. A score of 1 is given to each positive item and a score of 0 to each negative item. Scores ≥4/10 are considered indicative of neuropathic pain.

ID pain is a six-item self-questionnaire whose score ranges from 0 to 5 with higher scores corresponding to neuropathic pain or mixed pain with neuropathic component. The presence of pain limited to the joints (that is nociceptive pain) is scored minus 1.

All the questionnaires were scored as recommended by the developers [4, 6–8].

DN4 was filled in by the examiner; ID pain and I-NPSI, being self-administered questionnaires, were filled in by the patient.

Help in filling in questionnaires was provided when requested by the patients (e.g. visual problems, etc.). Problems in understanding some questions were recorded.

I-NPSI was filled in by the patient at the end of the clinical visit. However the result of clinical examination was not reported to the patient until I-NPSI was completed, to avoid bias.

The test–retest reliability was assessed in a subgroup of 25 clinically stable patients by administering the same protocol (DN4, ID pain, and I-NPSI), by the same examiner, after 3–5 days. Sensitivity to change was assessed in 32 patients whose symptoms changed after treatment, by administering the same protocol (DN4, ID pain, and I-NPSI), by the same examiner, after 3–6 weeks.

Statistical analysis

Demographic features and I-NPSI scores found in our sample of patients were compared with those of the study by Buohassira et al. [4], by one sample *t* test.

We used the Spearman's rank correlation coefficient for the correlation of I-NPSI score and subscores with VAS, ID pain, and DN4 (STAT-SOFT, OK, USA).

The test–retest reliability was tested by the Spearman–Browns test. To assess the sensitivity to change, we analyzed the correlation between changes of the I-NPSI scores and changes of the other measures (VAS, ID pain, DN4) by the Spearman's rank correlation coefficient. In the original

Table 3 Pain scores according to the used measurements (NPSI, VAS, DN4, and ID pain)

	Mean	Min value	Max value	Standard deviation
Q1	2.5	0	10.0	3.1
Q2	2.0	0	10.0	2.7
Q3	2.1	0	10.0	2.7
Q4	3.1	0	5.0	1.6
Q5	2.2	0	10.0	3.0
Q6	1.3	0	10.0	2.5
Q7	4.0	0	5.0	1.3
Q8	1.6	0	10.0	2.7
Q9	2.0	0	10.0	2.9
Q10	1.4	0	10.0	2.6
Q11	2.7	0	10.0	3.0
Q12	4.2	0	10.0	3.4
Burning (superficial)spontaneous pain	2.5	0	10.0	3.1
Pressing (deep)spontaneous pain	2.1	0	10.0	2.5
Paroxysmal pain	1.8	0	9.0	2.3
Evoked pain	1.7	0	10.0	2.2
Paresthesia/dysesthesia	3.5	0	10.0	2.8
I-NPSI total score	11.5	0	41.7	9.3
FW-NPSI	2.4	0	5	2.0
VAS (mm)	37.2	0	100.0	29.3
DN4 1–1	0.4	0	1.0	0.5
DN4 1–2	0.2	0	1.0	0.4
DN4 1–3	0.4	0	1.0	0.5
DN4 2–4	0.7	0	1.0	0.5
DN4 2–5	0.5	0	1.0	0.5
DN4 2–6	0.6	0	1.0	0.5
DN4 2–7	0.1	0	1.0	0.3
DN4 3–8	0.6	0	1.0	0.5
DN4 3–9	0.4	0	1.0	0.5
DN4 4–10	0.2	0	1.0	0.4
Total score DN4	4.2	0	10.0	2.7
ID pain				
Pins and needles	0.6	0	1.0	0.5
Hot/burning	0.6	0	1.0	0.5
Numb	0.7	0	1.0	0.5
Electric shocks	0.5	0	1.0	0.5
Touch of clothing	0.3	0	1.0	0.4
Joint	0.1	0	1.0	0.4
Total score ID Pain	2.4	−1	5.0	1.5

work by Bouhassira et al. factor analysis was performed to determine whether the ten items of the scale could be summarized into independent factors representing different dimensions of neuropathic pain: that was the way the five clinical dimensions were obtained. We performed a confirmatory factor analysis (that is an extension of factor analysis sometimes tested as a follow-up to the factor analysis procedure). In this case it was used to evaluate if, also in the Italian version, the subgroups identified in the original study were not redundant.

A P value <0.05 was considered significant.

Results

No significant difference on age and gender was found between the Italian and the French patient groups (Table 1).

Translation of the NPSI to Italian was accomplished and the back-translation to Italian corresponded to the original French and English versions. The mean duration for filling in the questionnaire was 6.5 ± 3.8 min (range 2–20) and it correlated with age ($P < 0.0006$, R 0.18). With every additional 10 years of age, the time for filling in the questionnaire increased by 1.8 min (R 0.18). Rarely, questions needed explanations (Q4 7 times, Q2 and Q12 6 times, and Q8 5 times).

Mean values, range, and standard deviations of VAS, DN4 and ID pain scores are reported in Table 3. As shown in Table 4, I-NPSI scores significantly correlated with the other pain scores. Table 5 shows the data concerning the relationship between changes to assess responsiveness: the NPSI changes significantly correlated with DN4, VAS, and ID pain changes. The test–retest reliability evaluation showed a high agreement between the I-NPSI scores of the two visits ($P = 0.001$).

Concerning the confirmatory factor analysis, each of the five dimensions of the NPSI corresponds to a relevant clinical component dimension of neuropathic pain and no redundancy was observed.

Discussion

During the last decade, patient-oriented questionnaires have gained great importance as outcome measures. These self-administered questionnaires are nowadays considered a necessary outcome measure for clinical trials and also in everyday clinical practice [9]. This is obviously, particularly true for pain assessment, where objective measurements are lacking.

Emerging evidence points to the importance of multi-center studies with sufficient sample size to allow reliable statistical analysis [10]. Thus, to increase the statistical power of clinical and research studies and to allow meta-analysis, the use of culturally equivalent standardized questionnaires as outcome measures is necessary. To enable the use of these questionnaires in languages and countries different from the ones in which they were developed, it is necessary to adapt them to the language and culture in which they are intended to be used.

Table 4 Construct validity: relationship between NPSI scores and VAS, DN4, and ID pain

	VAS	DN4	ID pain total score	ID pain subscore pins and needles	ID pain subscore hot/burning	ID pain subscore numb	ID pain subscore electric shocks	ID pain subscore touch of clothing	ID pain subscore Joint
Burning (superficial)	$P <$ E-15	$P <$ E-15	$P <$ E-15	$P = 0.0001$	$P <$ E-15	0.0006	NS	$P <$ E-5	NS
Spontaneous pain	$R = 0.5$	$R = 0.6$	$R = 0.5$	$R = 0.2$	$R = 0.7$	$R = 0.2$		$R = 0.3$	
Pressing (deep)	$P <$ E-15	$P <$ E-15	$P = 0.0004$	$P = 0.04$	$P = 0.0003$	NS	NS	$P = 0.02$	NS
Spontaneous pain	$R = 0.5$	$R = 0.4$	$R = 0.2$	$R = 0.1$	$R = 0.2$			$R = 0.1$	
Paroxysmal pain	$P <$ E-15	$P <$ E-15	$P <$ E-10	NS	$P = 0.03$	NS	$P <$ E-15	NS	NS
	$R = 0.5$	$R = 0.5$	$R = 0.4$		$R = 0.1$		$R = 0.6$		
Evoked pain	$P <$ E-15	$P <$ E-15	$P <$ E-15	$P = 0.00006$	$P <$ E-6	0.0001	$P = 0.03$	$P <$ E-15	NS
	$R = 0.5$	$R = 0.6$	$R = 0.5$	$R = 0.2$	$R = 0.3$	$R = 0.2$	$R = 0.1$	$R = 0.6$	
Paresthesia/ dysesthesia	$P <$ E-15	$P <$ E-15	$P <$ E-15	$P <$ E-15	$P = 0.0004$	$P <$ E-15	$P = 0.001$	$P = 0.0003$	NS
	$R = 0.6$	$R = 0.7$	$R = 0.6$	$R = 0.5$	$R = 0.2$	$R = 0.6$	$R = 0.2$	$R = 0.2$	
I-NPSI total score	$P <$ E-15	$P <$ E-15	$P <$ E-15	$P <$ E-8	$P <$ E-15	$P <$ E-8	$P <$ E-7	$P <$ E-10	NS
	$R = 0.8$	$R = 0.8$	$R = 0.6$	$R = 0.3$	$R = 0.5$	$R = 0.3$	$R = 0.3$	$R = 0.4$	
FW-NPSI	$P <$ E-15	$P <$ E-15	$P = 0.0005$	$P = 0.02$	$P = 0.0006$	NS	NS	$P = 0.01$	NS
	$R = -0.6$	$R = -0.5$	$R = -0.2$	$R = -0.1$	$R = -0.2$			$R = -0.1$	

R Spearman rank correlation, *P* *P* value, *NS* non significant

Table 5 Relationship between changes (differences-delta) in the NPSI scores and changes of the other subjective pain measures

	DELTA VAS	DELTA DN4	DELTA ID pain
Delta burning (superficial)	NS	$P = 0.001$	$P = 3$ E11
Spontaneous pain		$R = 0.5$	$R = 0.9$
Delta pressing (deep)spontaneous pain	NS	NS	NS
Delta paroxysmal pain	$P = 0.004$	$P = 0.01$	NS
	$R = 0.5$	$R = 0.4$	
Delta evoked pain	$P = 0.003$	$P = 0.0001$	NS
	$R = 0.5$	$R = -0.6$	
Delta paresthesia/dysesthesia	NS	$P = 0.002$	$P = 0.000006$
		$R = 0.5$	$R = 0.7$
Delta I-NPSI total score	NS	$P = 0.003$	$P = 0.000002$
		$R = 0.5$	$R = 0.7$
FW-NPSI	NS	$P = 0.002$	$P = 0.002$
		$R = 0.5$	$R = 0.7$

Being the study performed in a selected sample of patients with peripheral nervous system impairment, the results demonstrated that the Italian version of NPSI is valid and reliable to quantify neuropathic pain in patients with peripheral nerve diseases, and it is sensitive to changes due to treatment.

We used a methodology different from the one adopted by Bouhassira et al.: while they enrolled only patients with pain, we recruited consecutive patients regardless of the presence/absence of pain in order to verify if in patients without pain the NPSI would provide negative results. Moreover, we related the NPSI results to two different scales assessing neuropathic pain (while they used only a generic pain measure, the VAS). Interestingly, total I-NPSI score and subscores were not related to joint pain as measured by ID pain, since this domain concerns nociceptive involvement and not neuropathic involvement. Hence, although NPSI was not developed to discriminate between nociceptive and neuropathic pain, it may provide useful results for characterisation of pain/information on the characteristics of the pain. Our approach was able to show that I-NPSI is pain-specific (negative in case of absence of pain) and specific for neuropathic pain (higher correlations with the scales assessing neuropathic symptoms).

The time needed to fill-in the questionnaire as well as the correlation between the I-NPSI and VAS results obtained in our study were very similar to those reported in the original study.

The ability to detect symptom changes is crucial both for clinical practice and for clinical trial. NPSI questionnaire, the first validated tool to assess neuropathic pain in Italian language, provides clinicians with sub-categories of symptoms that are very useful, not only to better characterize neuropathic pain, but also to measure the response to therapy.

The new scores assessing temporal pattern of pain, NPSI-FW, showed high reliability having the same behavior as the other NPSI scores. Because the reduction of the frequency of pain episodes, besides the reduction of the pain intensity, is a goal of the therapy, this score may be useful to evaluate the efficacy of the therapy.

In conclusion, I-NPSI proved to have equivalent evaluation capacities in Italian population with peripheral nervous system involvement to the original one and NPSI-FW may represent an additional score that further improves the usefulness of NPSI.

Acknowledgments Other researchers involved are Aretini A (Siena), De Toni Franceschini L, Lopez I (Milano, San Raffaele), Francesca Gallia (Milano, Humanitas), Truini A (Roma, La Sapienza), Granata G, Caliandro P, Fusco F (Roma, Cattolica, Don Gnocchi), Iodice R (Napoli), Lucchetta M, Dalla Torre C (Padova), Negro E, Grimaldi S (Torino, S Giovanni Battista), and Pastorelli F (Bologna)

Conflict of interest statement The authors report no conflicts of interest.

Appendix I: Italian version of NPSI

Data:
Nome: Cognome:
Sesso:
Età:

Lei soffre di un dolore dovuto a un incidente o a una malattia del sistema nervoso. Questo dolore può essere di diversi tipi. Lei potrebbe provare un dolore spontaneo, ad esempio un dolore in assenza di qualsiasi stimolazione, che potrebbe essere continuo o manifestarsi sotto forma di brevi attacchi di dolore. Potrebbe anche provare un dolore provocato o accentuato dallo sfioramento, dalla pressione o dal contatto della parte dolorante con il freddo. Lei può provare uno o più tipi di dolore. Questo questionario è stato costruito per aiutare il suo medico a valutare e curare meglio i diversi tipi di dolore che prova.

Vorremmo sapere se ha dolore spontaneo, cioè dolore in assenza di stimolazioni. Per ciascuna delle seguenti domande, la preghiamo di scegliere il numero che descrive meglio *l'intensità media del dolore spontaneo che ha provato nelle ultime 24 ore*. Scelga il numero 0 se non ha provato questo tipo di dolore (faccia un cerchietto attorno a un solo numero).

Q1. Il dolore assomiglia a una sensazione di bruciore?

Nessuna sensazione di bruciore	0	1	2	3	4	5	6	7	8	9	10	La peggiore sensazione di bruciore che si possa immaginare

Q2. Il dolore assomiglia ad una stretta?

Nessuna stretta	0	1	2	3	4	5	6	7	8	9	10	La stretta più forte che si possa immaginare

Q3. Il dolore assomiglia a una sensazione di compressione?

Nessuna sensazione di compressione	0	1	2	3	4	5	6	7	8	9	10	La peggiore sensazione di compressione che si possa immaginare

Q4. *Nelle ultime 24 ore*, il dolore spontaneo è stato presente:
Scelga *la risposta* che descrive meglio il suo caso.

In continuazione	|__|
Da 8 a 12 ore	|__|
Da 4 a 7 ore	|__|
Da 1 a 3 ore	|__|
Meno di 1 ora	|__|

Vorremmo sapere se ha brevi attacchi di dolore. Per ciascuna delle seguenti domande, la preghiamo di scegliere il numero che descrive meglio *l'intensità media dei suoi attacchi di dolore nelle ultime 24 ore*. Scelga il numero 0 se non ha provato questo tipo di dolore (faccia un cerchietto attorno a un solo numero).

Q5. Il dolore è simile a delle scosse elettriche?

Nessun dolore simile a scosse elettriche	0	1	2	3	4	5	6	7	8	9	10	Il peggiore dolore simile a scosse elettriche che si possa

												immaginare
Q6. Il dolore è simile a una pugnalata?												
Nessun dolore simile a una pugnalata	0	1	2	3	4	5	6	7	8	9	10	Il peggiore dolore simile a una pugnalata che si possa immaginare
Q7. *Nelle ultime 24 ore*, quanti di questi attacchi di dolore ha avuto? Scelga *la risposta* che descrive meglio il suo caso.												
Più di 20												I__I
Da 11 a 20												I__I
Da 6 a 10												I__I
Da 1 a 5												I__I
Nessun attacco di dolore												I__I
Vorremmo sapere se avverte dolore provocato o accentuato dallo sfioramento, dalla pressione o dal contatto della parte dolorante con il freddo o con il caldo. Per ciascuna delle seguenti domande, la preghiamo di scegliere il numero che descrive meglio *l'intensità media del dolore provocato nelle ultime 24 ore*. Scelga il numero 0 se non ha provato questo tipo di dolore (faccia un cerchietto attorno a un solo numero).												
Q8. Il dolore è provocato o accentuato dallo sfioramento della parte dolorante?												
Nessun dolore	0	1	2	3	4	5	6	7	8	9	10	Il peggiore dolore che si possa immaginare
Q9. Il suo dolore è provocato o accentuato dalla pressione sulla parte dolorante?												
Nessun dolore	0	1	2	3	4	5	6	7	8	9	10	Il peggiore dolore che si possa immaginare
Q10. Il dolore è provocato o accentuato dal *contatto* della parte dolorante con il freddo?												
Nessun dolore	0	1	2	3	4	5	6	7	8	9	10	Il peggiore dolore che si possa immaginare
Vorremmo sapere se ha delle sensazioni insolite *nella parte dolorante*. Per ciascuna delle seguenti domande, la preghiamo di scegliere il numero che descrive meglio *l'intensità media delle sensazioni insolite nelle ultime 24 ore*. Scelga il numero 0 se non ha avuto questo tipo di sensazione. Q11. Ha una sensazione di aghi o spilli?												
Nessuna sensazione di aghi o spilli	0	1	2	3	4	5	6	7	8	9	10	La peggiore sensazione di aghi o spilli che si possa immaginare
Q12. Avverte un formicolio?												
Nessun formicolio	0	1	2	3	4	5	6	7	8	9	10	Il peggiore formicolio che si possa immaginare

References

1. Amadio PC (1993) Outcomes measurements. J.Bone Joint Surg Am 75:1583–1584
2. Tonali P, Padua L, Sanguinetti C, Padua R, Romanini E, Amadio P (1999) Outcome research and patient-oriented measures in the multiperspective assessment of neurological and musculoskeletal disorders. Consensus conference: third Roman neurophysiology day, outcome research in neurology and in musculoskeletal disorders—24 October 1998. Ital J Neurol Sci 20:139–140
3. Ware JE Jr, Sherbourne CD (1992) The MOS 36-item short-form health survey (SF-36). I. Conceptual framework and item selection. Med Care 30:473–483
4. Bouhassira D, Attal N, Fermanian J, Alchaar H, Gautron M, Masquelier E, Rostaing S, Lanteri-Minet M, Collin E, Grisart J,

Boureau F (2004) Development and validation of the neuropathic pain symptom inventory. Pain 108:248–257

5. Guillemin F (1995) Cross-cultural adaptation and validation of health status measures. Scand J Rheumatol 24:61–63
6. Bouhassira D, Attal N, Alchaar H, Boureau F, Brochet B, Bruxelle J, Cunin G, Fermanian J, Ginies P, Grun-Overdyking A, Jafari-Schluep H, Lantéri-Minet M, Laurent B, Mick G, Serrie A, Valade D, Vicaut E (2005) Comparison of pain syndromes associated with nervous or somatic lesions and development of a new neuropathic pain diagnostic questionnaire (DN4). Pain 114:29–36
7. Portenoy R (2006) Development and testing of a neuropathic pain screening questionnaire: ID pain. Curr Med Res Opin 22:1555–1565
8. Deschamps M, Band PR, Coldman AJ (1988) Assessment of adult cancer pain: shortcomings of current methods. Pain 32:133–139
9. Guyatt GH, Feeny DH, Patrick DL (1993) Measuring health related quality of life. Ann Intern Med 118:622–629
10. Freedman KB, Back S, Bernstein J (2000) Sample size and statistical power of randomised, controlled trials in orthopaedics. J Bone Joint Surg Br 83:397–402
