# Supplementary material for: Role of Sport Activity on Quality of Life in Charcot-Marie-Tooth 1A Patients
Source: J Clin Med. 2022 Nov 28;11(23):7032. doi: 10.3390/jcm11237032 (PMC9740468; doi:10.3390/jcm11237032)
Supplement: Supplementary file 1 [file jcm-11-07032-s001.zip › SELF-ESTEEM_ROSENBERG.pdf]

SELF-ESTEEM SCALE DI ROSENBERG

Prezza, M., Trombaccia, F. R., & Armento, L. (1997)

Istruzioni: La presente scala consiste in 10 affermazioni. Per favore legga attentamente le affermazioni e per ognuna di esse scelga quella che meglio descrive i sentimenti nutriti verso la sua persona. Per favore indichi quanto è d'accordo con ciascuna affermazione.

Perfettamente d'accordo
D'accordo
Non molto d'accordo
Disaccordo

	**Perfettamente d'accordo**	**D'accordo**	**Non molto d'accordo**	**dicaccordo**
1. Nell'insieme sono contento/a di me				
2. A volte penso di non valere niente				
3. Ritengo di avere un certo numero di pregi				
4. Sono capace di conseguire successi, con la maggior parte della gente				
5. Ho l'impressione che non ci sia molto in me di cui andar fiero/a				
6. A volte mi sento veramente inutile				
7. Penso di valere almeno quanto gli altri				
8. Mi piacerebbe poter avere più rispetto per me stesso/a				
9. Tutto considerato penso di essere un/a fallito/a				
10. Ho un'opinione positiva di me stesso/a				

Scoring:
Items 2, 5, 6, 8, 9 are reverse scored. Give "Strongly Disagree" 1 point, "Disagree" 2 points, "Agree" 3 points, and "Strongly Agree" 4 points. Sum scores for all ten items. Keep scores on a continuous scale. Higher scores indicate higher self-esteem

- 10-16 Bassa stima di Sé
- 17-33 Media stima di Sé
- 34-40 Alta stima di Sé

Bibliografia
Prezza, M., Trombaccia, F. R., & Armento, L. (1997). La scala dell'autostima di Rosenberg: Traduzione e validazione italiana. Bollettino di Psicologia Applicata, 223, 35-44.
