# Supplementary material for: Role of Sport Activity on Quality of Life in Charcot-Marie-Tooth 1A Patients
Source: J Clin Med. 2022 Nov 28;11(23):7032. doi: 10.3390/jcm11237032 (PMC9740468; doi:10.3390/jcm11237032)
Supplement: Supplementary file 1 [file jcm-11-07032-s001.zip › SF36 Questionario.pdf]

QUESTIONARIO
SULLO STATO DI SALUTE

SF-36 (V1) STANDARD

12/94
IQOLA SF-36 Italian Version 1.6

Copyright © New England Medical Center Hospitals, Inc., 1992
All rights reserved.
(IQOLA SF-36 Italian version 1.6)

QUESTIONARIO SULLO STATO DI SALUTE SF-36

Data consegna I_I_I I_I_I I_I_I N° codice paziente I_I_I_I_I_I

ISTRUZIONI: Questo questionario intende valutare cosa Lei pensa della Sua salute. Le informazioni raccolte permetteranno di essere sempre aggiornati su come si sente e su come riesce a svolgere le Sue attività consuete.

Risponda a ciascuna domanda del questionario indicando la Sua risposta come mostrato di volta in volta. Se non si sente certo della risposta, effettui la scelta che comunque Le sembra migliore.

1. In generale, direbbe che la Sua salute è:

(Indichi un numero)

Eccellente ………………………………………………………… 1
Molto buona ……………………………………………………… 2
Buona …………………………………………………………….. 3
Passabile ………………………………………………………….. 4
Scadente …………………………………………………………... 5

2. <u>Rispetto ad un anno fa</u>, come giudicherebbe, ora, la Sua salute in generale?

(Indichi un numero)

Decisamente migliore adesso rispetto ad un anno fa ………………..… 1
Un po' migliore adesso rispetto ad un anno fa ..……………………… 2
Più o meno uguale rispetto ad un anno fa ……………………………... 3
Un po' peggiore adesso rispetto ad un anno fa ………………………... 4
Decisamente peggiore adesso rispetto ad un anno fa ………………….. 5

Copyright © New England Medical Center Hospitals, Inc., 1992
All rights reserved.
(IQOLA SF-36 Italian version 1.6)

3. Le seguenti domande riguardano alcune attività che potrebbe svolgere nel corso di una qualsiasi giornata. La Sua salute La limita attualmente nello svolgimento di queste attività?

(Indichi per ogni domanda il numero 1, 2, o 3)

	**SI, mi limita parecchio**	**SI, mi limita parzialmente**	**NO, non mi limita per nulla**
a. **Attività fisicamente impegnative**, come correre, sollevare oggetti pesanti, praticare sport faticosi	1	2	3
b. **Attività di moderato impegno fisico**, come spostare un tavolo, usare l'aspirapolvere, giocare a bocce o fare un giretto in bicicletta	1	2	3
c. Sollevare o portare le borse della spesa	1	2	3
d. Salire **qualche** piano di scale	1	2	3
e. Salire **un** piano di scale	1	2	3
f. Piegarsi, inginocchiarsi o chinarsi	1	2	3
g. Camminare **per un chilometro**	1	2	3
h. Camminare **per qualche centinaia di metri**	1	2	3
i. Camminare **per circa cento metri**	1	2	3
l. Fare il bagno o vestirsi da soli	1	2	3

4. Nelle ultime 4 settimane, ha riscontrato i seguenti problemi sul lavoro o nelle altre attività quotidiane, a causa della Sua salute fisica?

Risponda SI o NO a ciascuna domanda

(Indichi per ogni domanda il numero 1 o 2)

	**SI**	**NO**
a. Ha ridotto **il tempo** dedicato al lavoro o ad altre attività	1	2
b. Ha **reso** meno di quanto avrebbe voluto	1	2
c. Ha dovuto limitare alcuni **tipi** di lavoro o di altre attività	1	2
d. Ha avuto **difficoltà** nell'eseguire il lavoro o altre attività (ad esempio, ha fatto più fatica)	1	2

Copyright © New England Medical Center Hospitals, Inc., 1992
All rights reserved.
(IQOLA SF-36 Italian version 1.6)

5. Nelle ultime 4 settimane, ha riscontrato i seguenti problemi sul lavoro o nelle altre attività, a causa del Suo stato emotivo (quale il sentirsi depresso o ansioso)?

Risponda SI o NO a ciascuna domanda

(Indichi per ogni domanda il numero 1 o 2)

	**SI**	**NO**
a. Ha ridotto **il tempo** dedicato al lavoro o ad altre attività	1	2
b. Ha **reso** meno di quanto avrebbe voluto	1	2
c. Ha avuto un calo di **concentrazione** sul lavoro o in altre attività	1	2

6. Nelle ultime 4 settimane, in che misura la Sua salute fisica o il Suoi stato emotivo hanno interferito con le normali attività sociali con la famiglia, gli amici, i vicini di casa, i gruppi di cui fa parte?

(Indichi un numero)

Per nulla ..…………………………………………………………… 1

Leggermente ………………………………………………………… 2

Un po' ……………………………………………………………….. 3

Molto ….…………………………………………………………….. 4

Moltissimo ………………………………………………………….... 5

7. Quanto dolore fisico ha provato nelle ultime 4 settimane?

(Indichi un numero)

Nessuno ...…………………………………………………………… 1

Molto lieve ..………………………………………………………… 2

Lieve ………………………………………………………………... 3

Moderato …..………………………………………………………… 4

Forte ……..………………………………………………………..... 5

Molto forte …………………………………………………………... 6

Copyright © New England Medical Center Hospitals, Inc., 1992
All rights reserved.
(IQOLA SF-36 Italian version 1.6)

8. Nelle ultime 4 settimane, in che misura il dolore L'ha ostacolata nel lavoro che svolge abitualmente (sia in casa sia fuori casa)?

(Indichi un numero)

Per nulla ..………………………………………………………… 1

Molto poco ..……………………………………………………… 2

Un po' …………………………………………………………….. 3

Molto …..…..……………………………………………………… 4

Moltissimo ………………………………………………………... 5

9. Le seguenti domande si riferiscono a come si è sentito nelle ultime 4 settimane. Risponda a ciascuna domanda scegliendo la risposta che più si avvicina al Suo caso. Per quanto tempo nelle ultime 4 settimane si è sentito…

(Indichi un numero per ogni domanda)

	**Sempre**	**Quasi sempre**	**Molto tempo**	**Una parte del tempo**	**Quasi mai**	**Mai**
a. vivace brillante?	1	2	3	4	5	6
b. molto agitato?	1	2	3	4	5	6
c. così giù di morale che niente avrebbe potuto tirarLa su?	1	2	3	4	5	6
d. calmo e sereno?	1	2	3	4	5	6
e. pieno di energia?	1	2	3	4	5	6
f. scoraggiato e triste?	1	2	3	4	5	6
g. sfinito?	1	2	3	4	5	6
h. felice?	1	2	3	4	5	6
i. stanco?	1	2	3	4	5	6

Copyright © New England Medical Center Hospitals, Inc., 1992
All rights reserved.

10. Nelle ultime 4 settimane, per quanto tempo la Sua salute fisica o il Suo stato emotivo hanno interferito nelle Sue attività sociali, in famiglia, con gli amici?

(Indichi un numero)

Sempre ....………………………………………………………	1
Quasi sempre ...………………………………………………………	2
Una parte del tempo …...……………………………………………..	3
Quasi mai ..……………………………………………………………..	4
Mai ……...…………………………………………………………...	5

11. Scelga la risposta che meglio descrive quanto siano **VERE** o **FALSE** le seguenti affermazioni.

(Indichi un numero per ogni affermazione)

	**Certamente vero**	**In gran parte vero**	**Non so**	**In gran parte falso**	**Certamente falso**
a. Mi pare di ammalarmi un po' più facilmente degli altri	1	2	3	4	5
b. La mia salute è come quella degli altri	1	2	3	4	5
c. Mi aspetto che la mia salute andrà peggiorando	1	2	3	4	5
d. Godo di ottima salute	1	2	3	4	5

Copyright © New England Medical Center Hospitals, Inc., 1992
All rights reserved.
